# The Effects of ICT Use on Depression in Older Adults: The Mediating Role of Social Participation

**DOI:** 10.1093/geroni/igaf122.3059

**Published:** 2025-12-31

**Authors:** Mina Hwang, Yeji Hwang

**Affiliations:** Seoul National University, Seoul, Republic of Korea; Seoul National University, Seoul, Republic of Korea

## Abstract

As information and communication technologies (ICTs) become integral to daily life, their role in reducing depression among community-dwelling older adults has gained attention. However, the underlying mechanisms remain unclear. As one possible mechanism is via social participation, this study aimed to examine the effects of ICT usage on depression and whether social participation mediates the relationship. This study was a secondary data analysis using the National Health and Aging Trends Study (NHATS) Round 13 (N = 6,722). Participants were categorized into 4 types of ICT usage: Non-use, CT-use, IT-use, and ICT use. Social participation was classified as formal and informal participation. For data analysis, multiple parallel mediation analyses were conducted. Findings revealed that both formal (B=-0.098, 95%CI=-0.148,-0.053) and informal (B=-0.139, 95%CI=-0.188,-0.093) social participation had an indirect effect on the relationship between ICT use and depression. Additionally, ICT use had a direct effect on depression (B=-0.491, 95%CI=-0.701,-0.280). When examining CT use, both formal (B=-0.041, 95%CI=-0.069,-0.019) and informal (B=-0.093, 95%CI=-0.133,-0.058) social participation exhibited an indirect effect on depression. However, there was no direct effect of CT use on depression (B=-.183, 95%CI=-0.428,0.063). For IT use, only informal social participation (B =-0.060, 95%CI=-0.098,-0.029) had an indirect effect on depression, while no direct effect of IT use on depression was observed (B=-0.109, 95%CI=-0.424,0.206). These findings suggest ICT use mitigates depression through social connections, though effects vary by types of ICT use. A comprehensive approach is needed when designing ICT-based interventions. Such intervention should promote social participation to maximize their effectiveness to reduce depression among older adults.

